# Pharmacies and use of antibiotics: a cross sectional study in 19 Arab countries

**DOI:** 10.1186/s13756-024-01458-6

**Published:** 2024-09-18

**Authors:** Hadeer Hafez, Mohamed Saad Rakab, Adham Elshehaby, Ahmed Ibrahim Gebreel, Mohamed Hany, Mohammad BaniAmer, Mona Sajed, Sara Yunis, Sondos Mahmoud, Marwan Hamed, Maha Abdellatif, Aseel Nabeel Alomari, Amr Esam Moqbel, Omnia Samy El-Sayed, Mohamed Elshenawy, Mohamed Tolba, Muhammad Saeed

**Affiliations:** 1 Faculty of Medicine, October 6th University, 262, 7th Dis, October 6th City, Giza, Egypt; 2 Faculty of Medicine, Mansoura University, Dakahlia, Egypt; 3 Faculty of Pharmacy, October 6th University, Giza, Egypt; 4 Faculty of Medicine, Zagazig University, Al Sharqia, Egypt; 5 Faculty of Medicine, Jordan University of Science and Technology, Irbid, Jordan; 6 Faculty of Pharmacy, AlAzhar University for Girls, Cairo, Egypt; 7 Faculty of Dentistry, AlAzhar University, Gaza, Palestine; 8 Faculty of Applied Medical Science, Jordan University of Science and Technology, Irbid, Jordan; 9 Faculty of Pharmacy, American University in Madaba, Amman, Jordan; 10 Faculty of Medicine, Assiut University, Assiut, Egypt; 11 Faculty of Pharmacy, Yarmouk University, Irbid, Jordan; 12 Faculty of Medicine and Health science, Aden University, Aden, Yemen; 13 Faculty of Medicine, Misr University For Science And Technology, Giza, Egypt; 14 Head of Internal Medicine Department, October 6th University, Giza, Egypt

**Keywords:** Antibiotics, Pharmacists, Antimicrobial resistance, Bacterial resistance, Antimicrobial stewardship

## Abstract

**Background:**

The rise of antimicrobial resistance, which is partially attributed to the overuse and/or misuse of antibiotics in health care, is one of the world’s largest public health challenges. The distribution of antibiotics in absence of a prescription in pharmacies is a significant contributor to the growing global public health crisis of antibiotic resistance. A pharmacist’s clinical and lawful knowledge of antibiotic provide has an impact on the proper way to dispense medication. There are few novel studies assessing pharmacists comprehension and experience in prescribing antibiotics in low- and middle-income countries, including those in the Arabian region.

**Objectives:**

(I) assess pharmacy team members Knowledge about antibiotics as reported by individuals themselves and their behavior in dispensing antimicrobial without a prescription and (ii) find potential influences on this behavior.

**Setting:**

Pharmacists were chosen from various regions in Algeria, Bahrain, Egypt, Iraq, Jordan, Kuwait, Lebanon, Libya, Morocco, Oman, Palestine, Qatar, Saudi Arabia, Somalia, Sudan, Syria, Tunisia, the United Arab Emirates, and Yemen, based on their convenience and ease of access.

**Methods:**

A descriptive cross-sectional assessment among a random sample (*n* = 2833) of community pharmacists was conducted Utilizing a structured, validated, and questionnaire that underwent pilot testing, a comprehensive survey with four distinct sections covering biography, knowledge, practice, and attitude domains was employed.

**The main outcome:**

Measures were knowledge, attitude, and practice toward dispensing antibiotics without prescription.

**Results:**

Of the 3100 pharmacists reached, 2833 completed and return the questionnaires (response rate 91.3%). Most of the respondents were male (57.4%). Aged between 19 and 31 years old (76.2%). Most of them held a B.Sc. Degree (78.5%). Worked as staff pharmacists (73.2%). During the survey, it was discovered that there were gaps in their knowledge regarding antibiotic usage. A total of 45.7% of the respondents were unaware that antibiotics can be used as prophylaxis, while 33.3% did not recognize the consequences of making incorrect antibiotic choices. Regarding their practice patterns, 53.8% of the pharmacists admitted that they did not consistently adhere to guidelines when dispensing antibiotics. In terms of attitudes toward antibiotic usage, 36.8% disagreed with the guidelines of not supply antibiotics without a prescription, suggesting some variation in opinions among pharmacists on this matter. Additionally, a significant percentage (75%) believed that community pharmacists had qualifications to prescribe antibiotics for infections.

**Conclusion:**

The recent survey has shed light on the differences among pharmacists in regard to dispensing antibiotics without prescriptions and their understanding of resistance. The findings are concerning, indicating a deficient in of knowledge as regards the use of antibiotics. It is crucial to implement regulations and enhance education efforts to tackle the growing problem of resistance. Collaboration between healthcare professionals and awareness campaigns is essential in addressing this issue.

## Impact on practice

Community pharmacists may contribute to inappropriate antibiotic usage and microbial resistance by distributing antibiotics without a prescription.

It is crucial to develop and put into action a clear strategy to promote sensible antibiotic usage in a community pharmacy context.

It is advised that instructional tactics regarding the prescription of antibiotics and antibiotic resistance be improved.

To changing community pharmacists’ dispensing practices, educational programs emphasizing attitude adjustments and acknowledging the professional code of ethics are required.

Community pharmacists can modify patient behaviors and educate patients on the proper use of antibiotics.

## Introduction

Before the development of antibiotics, it was impossible to cure ultimately lethal bacterial illnesses including meningitis and bacteremia. Unfortunately, the development of antibiotic-resistant bacteria has been accelerated in recent decades due to the abuse and misuse of antibiotics as well as societal and economic issues, making pharmacological therapy less effective [1]. Nowadays, antibiotic resistance accounts for at least 700,000 annual deaths worldwide anti-microbial resistance (AMR). The World Health Organization (WHO) estimates that without new and improved treatments, this figure might increase to 10 million by 2050, emphasizing a serious health issue [1]. overuse of antibiotics is the major cause of AMR. Following the adoption of mobile genetic materials like plasmids, the resistant bacteria often grow by mutations or, more frequently, by acquiring resistance genes already present in other bacteria [2]. Genes that are activated by exposure to clinical doses of antibiotics can also cause intrinsic resistance in bacteria [3]. Then, the antibiotic-resistant bacteria pass on their antibiotic-resistant genetic material to the non-resistant bacteria, which causes them to develop antibiotic resistance [4].

The emergence of bacterial strains that are resistant to antibiotics is a serious problem for the healthcare systems of many nations; it can be viewed because of antibiotic overuse [5]. By passing a ring of DNA to different species or strains, antibiotic-resistant bacteria can quickly spread throughout the human body. E. coli, P. aeruginosa, K. pneumoniae, S. aureus, and E. faecalis are the most prevalent and clinically significant bacterial species found in European hospitals, according to the European Center for Disease Prevention and Control (ECDC) [5].

Furthermore, the suitability of prescription practices is challenged, as antibiotics are often improperly utilized in cases of viral infections. In the United States, approximately 7–8% of cases entering the Emergency Department receive at least one antibiotic prescription daily [6]. also, abuse of antibiotics poses a serious health risk in the Middle East. Unfortunately, parents have unrestricted access to antibiotics at the pharmacy [7]. They believe that every case of an upper respiratory tract infection should be treated with antibiotics, even without consulting a doctor, based on flawed notions, which has led to an accepted cultural trend of antibiotic abuse [7]. People utilize them as a form of self-medication because they believe they are the greatest options. Additionally, as people start to feel better, they stop taking the antibiotics for the whole recommended duration [7].

In a study that described the contributors to antibiotic resistance among the general public and healthcare providers. Public misconceptions, access to antibiotics without prescriptions, and leftover antibiotic use contribute to irrational behaviors. Among healthcare providers, inadequate education, pharmaceutical promotion, and limited diagnostic tools influence antibiotic prescribing [8].

In contrast, pharmacists are vital members of the healthcare team who play a significant role in the use of medications and the provision of guidance on how to use medications appropriately [9]. Healthcare systems are under threat from antibiotic resistance on a worldwide scale [10]. The improper use of antibiotics, which includes improper use, misuse, abuse, and incorrect administration, is one of the main causes of this issue [10]. Prescriptions without antibiotics is a significant additional factor. Much research held pharmacists accountable for this wrongdoing as healthcare providers. As a part of the community, we should investigate community pharmacists’ understanding, beliefs, and actions regarding the prescription of antibiotics, antibiotic resistance, and antibiotic stewardship [10]. Community pharmacists, as primary care providers, are an underutilized resource in anti-microbial stewardship (AMS) [11]. Primary care plays an important role in tackling AMR as the principle of balancing access to antimicrobials while ensuring optimal use is agnostic to health setting [11]. An important area of research should be focused on how to understand the perception and practices of AMS involvement against AMR [11]. However, there is an opportunity to understand the sociological factors that influence the profession contribution to stewardship practice, particularly across a broader spectrum of sector stakeholders at the individual, practice, system, and policy levels [11].

The growing availability of antibiotics in nations where they are available without a prescription, allowing people to self-medicate without consulting a doctor, is a significant factor [12]. Additionally, it could be brought on by issues with money, ignorance, inadequate access to healthcare, or even a lack of time [12]. Therefore, Community pharmacy and prescription drug leftovers from prior prescriptions are the primary sources of antibiotics intended for self-medication [13]. This demonstrates that the uncontrolled practice of self-medication and subsequent antibiotic resistance are mostly caused by the dispensing of antibiotics without a formal prescription and a lack of awareness regarding the abuse of antibiotics [13]. Lack of strong regulatory enforcement mechanisms, customer pressure, time and financial costs, accessibility to community pharmacies, and patients’ trust in pharmacists are the most often cited factors of Dispensing Antibiotics without prescription (DAWP) [14].

To effectively minimize DAwP and its detrimental effects on public health, a multimodal strategy including educational interventions and improving the general people access to, and cost of healthcare facilities is needed [15]. First, it is important to aggressively enforce national laws limiting the selling of antibiotics [14, 15]. Second, instructional initiatives that improve community pharmacists’ adherence to the code of ethics and professionalism are needed. Finally, expanding public healthcare access and raising awareness of antibiotic abuse could also help to decrease the sale of antibiotics without a prescription [14, 15].

In contrast to the region under studying, in a cross-sectional study that was held in Saudi Arabia it was found that the prevalence of self-medication with antibiotics was found to be (77.5%) [16]. In Syria they revealed in a survey regarding antibiotic use, most respondents in a survey regarding antibiotic use, most respondents, 59.6% stated that they take antibiotics based on pharmacist advice only and 80% do not complete the full antibiotic course [17]. And in Jordan, another study found that out of the 457 antibiotics dispensed, almost one third were without prescription [18]. A simulated patient study in Yemen resulted that most pharmacies (73.3%) dispensed antibiotics without medical prescriptions in different levels of demand [19]. Furthermore, a cross-sectional survey in Egypt reported that nearly 67% of the pharmacists reported that patients were more likely to be given antibiotics for showing any sign or symptom of COVID-19 infection, and 82% of the dispensed antibiotics were given upon physician recommendation. As a result of that, antibiotics were dispensed heavily during this pandemic without proper clinical indication and for long durations supporting the idea of antibiotic misuse [20].

Pharmacists dispense these antibiotics without a prescription approved by a doctor, and this in itself is a big problem that causes the destruction of society year after year. Therefore, understanding of their knowledge, attitudes, and practice (KAP) in relation to public usage of antibiotics can greatly impact antibiotic-related issues. Hence, the significance of our study, which highlights the need for education programs and courses provided to pharmacists monitoring the prescription of antibiotics that should not be given without a prescription by a doctor, which in turn will increase awareness among patients so that antibiotics become vital is only in the hands of those who are in the best-known need for It. This study aimed to assess KAP pertaining to DAwP and to explore significant risk factors.

### Aim of the study

To draw attention to the role that pharmacists played in the development, spread, and management of AMR (anti-microbial resistance) in 19 Arab nations. This study, which examines community pharmacists’ knowledge, attitudes, and practices with relation to antibiotics, AMR (anti-microbial resistance), and antimicrobial stewardship, is the first of its kind to be carried out in all of these nations together. Hope that these findings will eventually shed light on AMR anti-microbial resistance) and provide pharmacists more ability to combat it. Additionally, it might help the decision makers create a strategic plan to undertake antimicrobial stewardship, enforce laws against prescription-free drug distribution, and enhance pharmacy national curriculum in these areas.

## Methods

### Study design

A descriptive cross-sectional survey among community pharmacists, using a structured and validated questionnaire. was conducted in a convenience sample of pharmacists in 19 Arab countries; Algeria, Bahrain, Egypt, Iraq, Jordan, Kuwait, Lebanon, Libya, Morocco, Oman, Palestine, Qatar, Saudi Arabia, Somalia, Sudan, Syria, Tunisia, United Arab Emirates, and Yemen. this work was carried out in accordance with the guidelines and recommendations of the Declaration of Helsinki and ethical approval was obtained for this study from the research ethics committee in the university October 6th University (Protocol approval Code: No. PRC-Me-2307002).

#### Eligibility criteria

This study included participating pharmacies’ owners, managers, or staff members who had at least bachelor’s degree in pharmacy studies. Participants was recruited by research collaborators in each country.Also, based on their availability and accessibility.

#### Exclusion criteria

Students who did not complete their studies in pharmacy school at the time of data collection.

#### Sampling and sample size

The study methodology involved convenience sampling. First, implementing random sampling requires a sample framework, which may not exist or be impossible to acquire in many countries (for example, a database of all pharmacies in each nation). By allowing researchers to include facilities they already know to be eligible, convenience sampling facilitates the conduct of the study. Second, no compensation for expenses was given to research collaborators because the current study is self-funded in local study settings. Convenience sampling allows for the recruitment of easily accessible facilities, reduces the need for travel to remote locations, and enables researchers to involve facilities with which they already have connections, saving research collaborators’ time and effort.

There were no limitations on the number of nations, locations, or participants in terms of sample size. We made a huge effort to include 19 countries from The Arabian nations and increase the homogeneity of the sample by predefining eligibility criteria for both settings and participants in order to reduce the limitations of the convenience-based sampling approach and increase the representativeness of the sample.

#### Questionnaire design

A four-part systematic survey consisting of biography, knowledge, practice and attitude domains was used.

A comprehensive literature search was performed of the electronic databases (PubMed), and relevant studies [15, 18, 21–23] were reviewed with the aim of informing the design of the questionnaire for the present study. The validity of the instrument and comparability with those surveys were crucial considerations in choosing which questions to include. This process led to the questionnaire internal reliability (Cronbach Alpha) to reach high level of internal reliability = 0.787. During the literature review, every attempt was made to verify content validity. Two academics with substantial backgrounds in survey research, along with two pharmacists, carried out the face and content validity. In light of the comments received, questions were rewritten, reorganized, and reformatted. The final draught included of thirty-nine questions, divided into four sections. The first section (seven items) gathered data on demographic characteristics of the pharmacists, including gender, age, educational level, major, years of experience, type of the pharmacy and the job status. The second section (nine items) evaluated community pharmacists’ knowledge about the usage and describing of antibiotics, the legal status of DAwP and its implications with regards to growing AMR (Anti-microbial resistance) and public health. For each question in this section, respondents were given options of ‘true’, ‘false’, and ‘don’t know’ to choose from. The third section consisted of six evaluated practices of community pharmacists towards DAwP and consisted of thirteen questions (e.g., prescription of AB for common cold, considering DAwP as a problem, number of antibiotics with/without prescription dispensed per day, asking about history, symptoms, drug reactions, ARDs and drug allergies, educate patient about the appropriate use of antibiotics and resistance-related issues, check if antibiotic prescriptions are prescribed in accordance with local guidelines before dispensing, what to advice patient with common cold symptoms, actions taken if patient asked about antibiotic without having a prescription and/or one blister of it, counselling about the dose and duration of treatment and the commonest antibiotics dispensed regularly).Finally, the fourth section consisted of ten questions that were designed to assess community pharmacists’ attitudes towards DAwP. Respondents were given options of ‘agree’, ‘disagree’, and ‘neutral’ to choose from. Respondents were also given a list of solutions to correct the antibiotic abuse problem to choose from.

### Data collection

#### Research collaborators

The research managing team of the current study is composed of the authors of the current manuscript, with the first author being the principal investigator. Due to the nature of the present study, the research managing team recruited collaborators in the 19 countries under investigation.

#### Data collection

A web survey used to gather data. The online survey was created in a way that needed minimal electronic skills to access, complete, and submit the questionnaire. Google Forms [24]was used to build the survey. A number of successive webpages were created with the survey items. Between screens, you are free to turn around. This layout, which resembles filling out a survey on paper, was created to stop users from scrolling through a long page and possibly getting lost. Research collaborators may distribute the survey links to study participants in any convenient way, such as facility intranet or personal e-mail. In addition, paper questionnaires were made available for use if necessary, taking into account the particular requirements of each site, such as restricted online access or lack of experience with online surveys.

#### Data management and analysis

Collected data was stored in an encrypted drive and is accessed only by the research management team to ensure data confidentiality. Spreadsheets and IBM SPSS Statistics (Version 26) predictive analytics software [25]was used for data coding, cleaning, and analysis. Descriptive analysis was used for this data. Categorical variables were presented as frequency and percentage while mean and standard deviation were used to describe continuous variables. Chi square test used to assess the association between respondent’s demographic data and knowledge score. P value less than 0.05 is defined as statistical significance.

### Sampling and data collection

A pretested, pre-validated structured and anonymous questionnaire was administered to 3200 community pharmacists from different areas of the 19 countries participating, together with informed consent to participate. Participants were selected based on their availability and accessibility, and there were no specific inclusion criteria. Research collaborators collected the questionnaire from respondents after completion on spot.

## Results

### Socio-demographic characteristics of study population

We approached 3100 pharmacies. Among them, 2833 completed and returned the questionnaire with a response rate of 91.3%. Community pharmacies formed the majority (71.2%) followed by hospital pharmacies (17.6%) and lastly, chain pharmacies (11.2%). The demographic and professional characteristics of the participants are detailed in Table 1.

**Table 1:** Demographic and professional characteristics of the participants

Characteristics		Frequency	%
***Gender***			
	Male	1626	57.4
	Female	1207	42.6
***Age***			
	19 - <25	1076	38.0
	25 - <31	1082	38.2
	31 - <40	530	18.7
	> 40	145	5.1
***Educational level***			
	B. Sc	2225	78.5
	Postgraduate study	608	21.5
***Major***			
	Pharmacy	2151	75.9
	Doctor of pharmacy	682	24.1
***Years of experience***			
	< 1 (trainee)	561	19.8
	1–2	653	23.0
	3–5	864	30.5
	6–10	393	13.9
	> 10	362	12.8
***Type of the pharmacy***			
	Hospital pharmacy	318	11.2
	Community pharmacy	2018	71.2
	Chain pharmacy	497	17.5
***Job status***			
	Staff pharmacist	2075	73.2
	Pharmacy manager	465	16.4
	Pharmacy owner	293	10.3

### Knowledge

**Table 2:** Knowledge of participants towards antibiotics use and resistance

Question	Having knowledge *n*(%)	No knowledge *n*(%)
*Antibiotics help treat common cold, cough, flu and fever (temperature < 38.5°)?*	1666 (58.8)	1167 (41.2)
*Antibiotics can be used to alleviate pain?*	2087 (73.7)	746 (26.3)
*Antibiotics can be used as prophylaxis?*	1538 (54.3)	1295 (45.7)
*Antibiotics can cause secondary infections after killing normal flora?*	2282 (80.6)	551 (19.4)
*Microorganisms can develop resistant to antibiotics, whereas human bodies cannot develop resistance to wards antibiotics?*	2059 (72.7)	774 (27.3)
*Antimicrobial resistance considered as global problem?*	1865 (65.8)	968 (34.2)
*Wrong choice of antibiotics may lead a pathogen to lose its sensitivity towards a specific antibiotic?*	1889 (66.7)	944 (33.3)
*Dispensing antibiotics without prescription (DAwP) is a common practice among community pharmacists in your country?*	2137 (75.4)	696 (24.6)
*Pharmacists can be penalized for (DAwP)?*	1301 (45.9)	1532 (54.1)

Nearly half of the pharmacists did not know that antibiotics can be used as prophylaxis (45.7%). A third of pharmacists (33.3%) did not know that wrong choice of antibiotics may lead a pathogen to lose its sensitivity towards a specific antibiotic. Almost a third of them (34.2%) did not consider antimicrobial resistance as a global problem. Also, an important question had a high percentage (41.2%) of wrong answers; “Antibiotics help treat common cold, cough, flu and fever (temperature < 38.5°)?”. More than half of the participants (54.1%) did not know that (DAwP) is illegal in Egypt. Details are shown in Table 2.

### Practice

**Table 3:** Practice of participants towards antibiotics use and resistance

Question	Optimal practice *n*(%)	Sub-optimal practice *n*(%)	Education level, *p*-value	Experience, *p*-value	Antibiotics dispensed/day, *p*-value
*Do you Ask doctor to prescribe antibiotics for common cold?*	1540 (54.3)	1293 (45.7)	0.122~	< 0.001*,~	< 0.001*,~
*Do you think there is any problem if you dispense medication without prescription?*	1938 (68.3)	899 (31.7)	< 0.001*,~	< 0.001*,~	0.839~
*I ask the patient’s history and symptoms of their infections before deciding to dispense antimicrobials*	1716 (60.6)	1117 (39.4)	0.006*,~	< 0.001*,~	< 0.001*,~
*Before I dispense an antibiotic, I seek additional clinical information like drug interactions, ADRs, allergy…*	1642 (58)	1191 (42)	< 0.001*,~	< 0.001*,~	< 0.001*,~
*I educate patients about the appropriate use of antibiotics and resistance-related issues*	1838 (64.9)	995 (35.1)	0.115~	< 0.001*,~	< 0.001*,~
*I check if antibiotic prescriptions are prescribed in accordance with local guidelines before dispensing*	1309 (46.2)	1524 (53.8)	< 0.001*,~	< 0.001*,~	< 0.001*,~

* p-value < 0.05, statistically significant association

~Results obtained by Chi-Square test

Table 3 shows the results of practice assessment. More than half of the pharmacists (53.8%) showed lack of adherence to following local guidelines before dispensing antibiotics. About 67.5% of the respondents agreed that refusing (DAwP) will negatively affect sales and profits. When asked about their advice for an adult patient suffering from a sore throat, runny nose and cough, the most frequent answer was “Symptomatic treatment using OTC medications” (64.3%), followed by “Refer to a doctor” (24.9%), the answer “treatment with antibiotics” was chosen by only 6%. Other details are shown in Table 3.

**Fig. 1:**
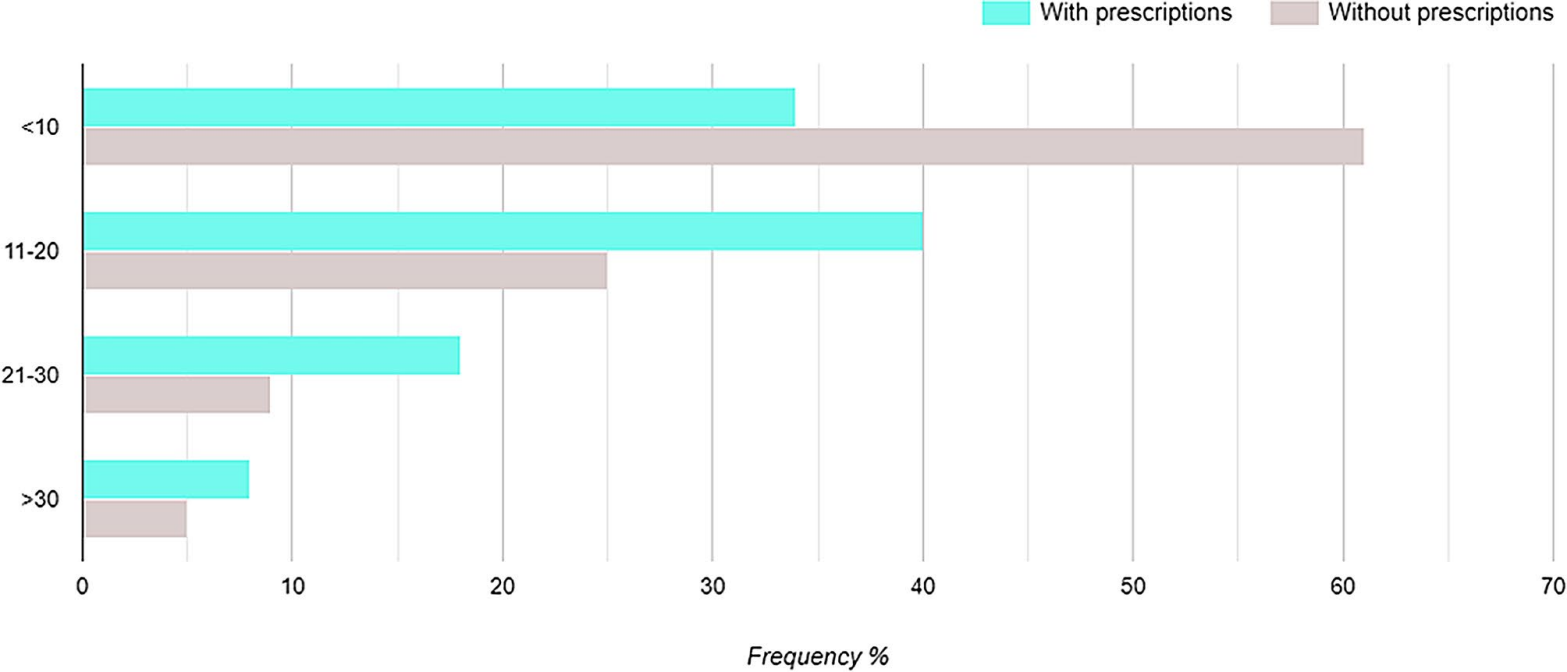
Number of antibiotics dispensed per day in participating pharmacies

We asked the pharmacists about their reaction when they are unsure about the appropriateness of the antibiotic in the patient’s case, 44.5% said that they always do communicate with prescribers and 20.5% said that they rarely find medical errors in prescriptions indicating good practice. On the other hand, 12.8% don’t communicate because of poor physicians’ responsiveness, 16.1% said that they correct errors by themselves without referring to the prescriber and 6.1% don’t have time to communicate with the prescribers. Numbers of antibiotics dispensed in the participating pharmacies are shown in Fig. 1.

**Fig. 2:**
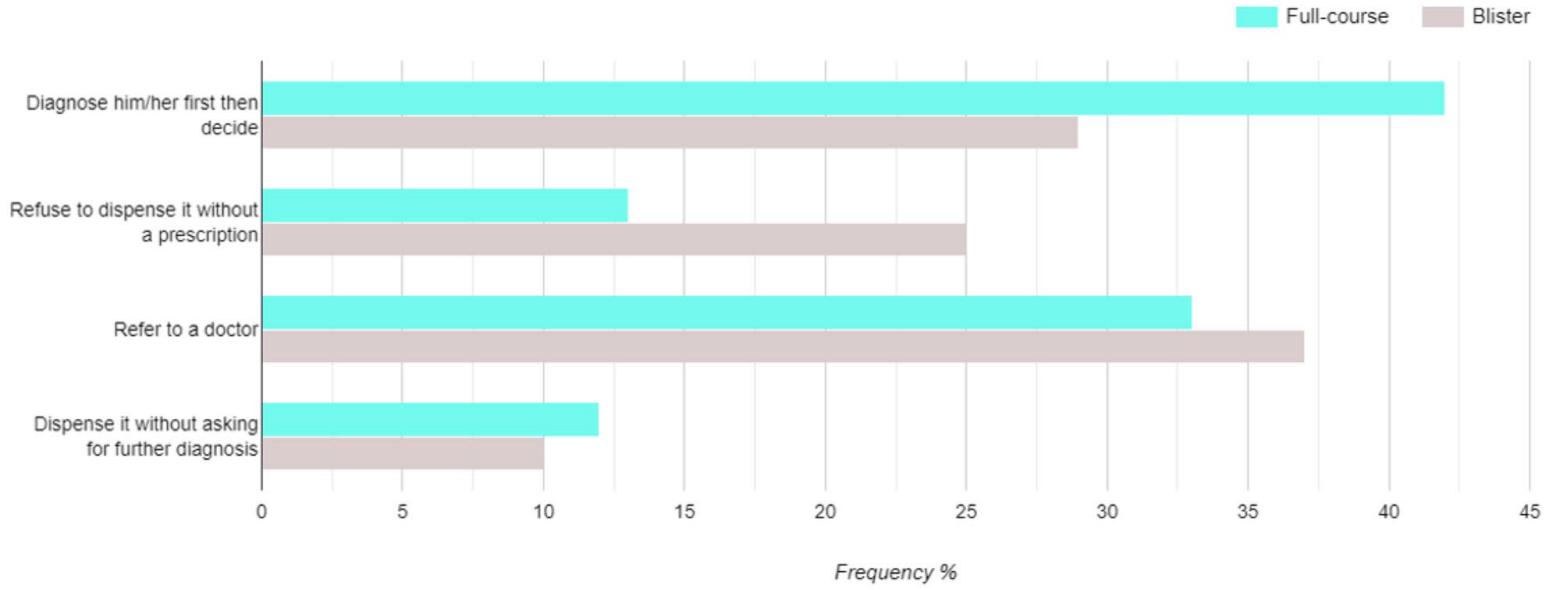
Pharmacists’ reactions when asked to dispense antibiotic without prescription

**Fig. 3:**
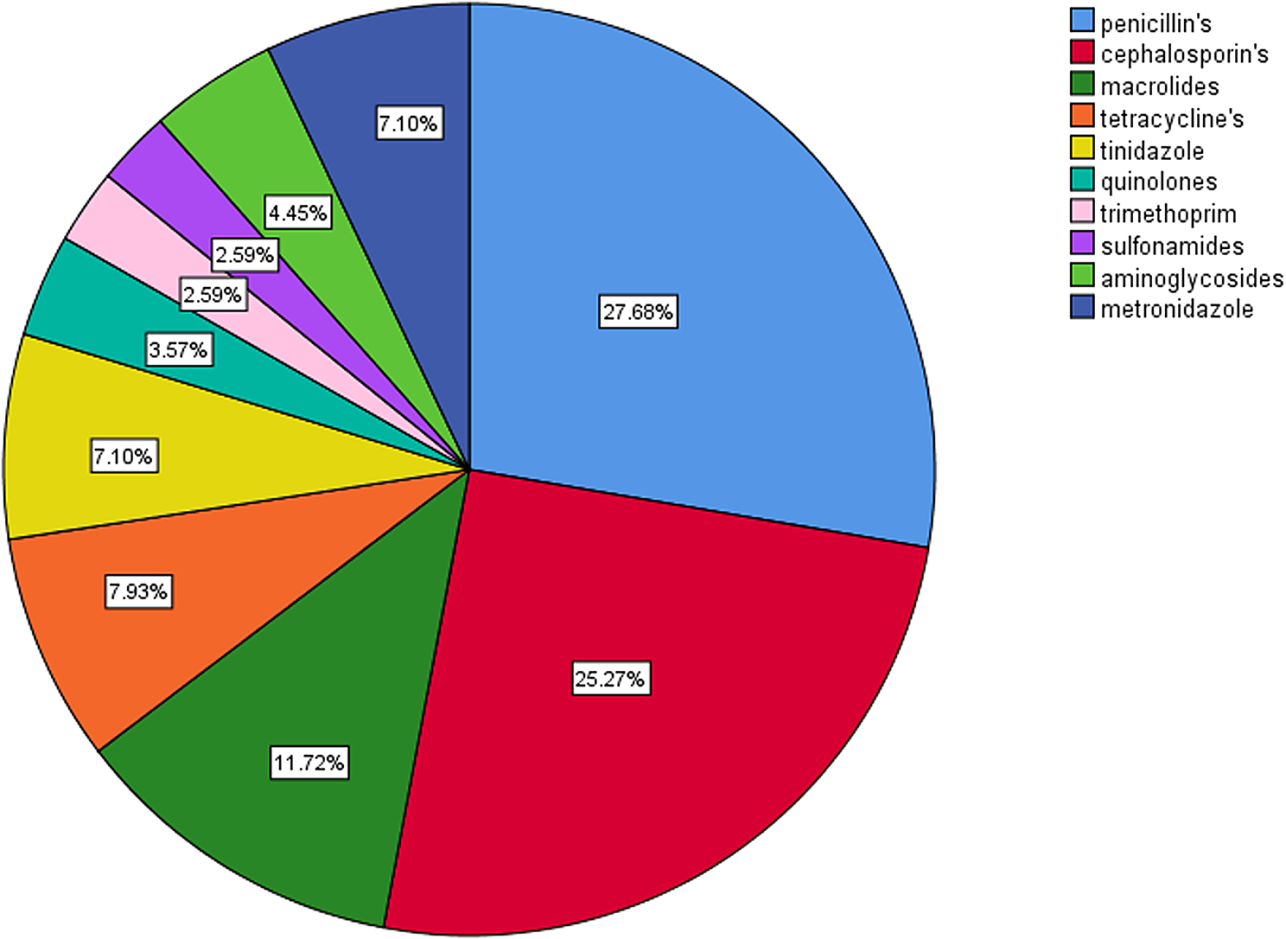
Type of antimicrobial group dispensed the most

The respondents showed more tendency to dispense antibiotics without prescription when the patients ask them for a full course (12.3%) compared with dispensing one blister (9.7%), answers are presented in Fig. 2. Penicillin and cephalosporins constituted more than half (53%) the antimicrobials dispensed, other antimicrobial agents are demonstrated in Fig. 3.

### Attitude

**Table 4:** Attitude of participants towards antibiotics use and resistance

Question	Correct attitude *n*(%)	Wrong attitude *n*(%)
*Do you agree with the policy of not dispensing antibiotics without prescription?*	1790 (63.2)	1043 (36.8)
*Does the fact that a patient is taking an antibiotic increases the risk of developing resistance?*	1941 (68.5)	892 (31.5)
*Does the patient have to terminate his/her treatment with the antibiotic once he/ she feels better?*	1860 (65.7)	973 (34.3)
*Can new antimicrobials development solve antimicrobial resistance problem?*	1307 (46.1)	1526 (53.9)
*Do you agree that antimicrobials are sometimes dispensed without medical prescription because the patient is known to have difficulty in obtaining a medical consultation?*	1538 (54.3)	1295 (45.7)
*Currently, community pharmacists are qualified to prescribe antibiotics to patients with bacterial infections (mild- moderate upper respiratory infections, skin infections, etc.), do you agree?*	696 (24.6)	2137 (75.4)
*Patients should be advised to keep part of the antibiotic course for another occasion, which will also be an added advantage for them in cutting down their medical costs, do you agree?*	1564 (44.2)	1269 (44.8)
*Can formal teaching on proper usage of antimicrobial agents among healthcare students minimize the phenomena of antibiotic resistance?*	1987 (70.1)	846 (29.9)
*Will refusing to dispense antibiotics without prescriptions negatively affect sales and profits of pharmacies?*	1557 (55)	1276 (45)

**Fig. 4:**
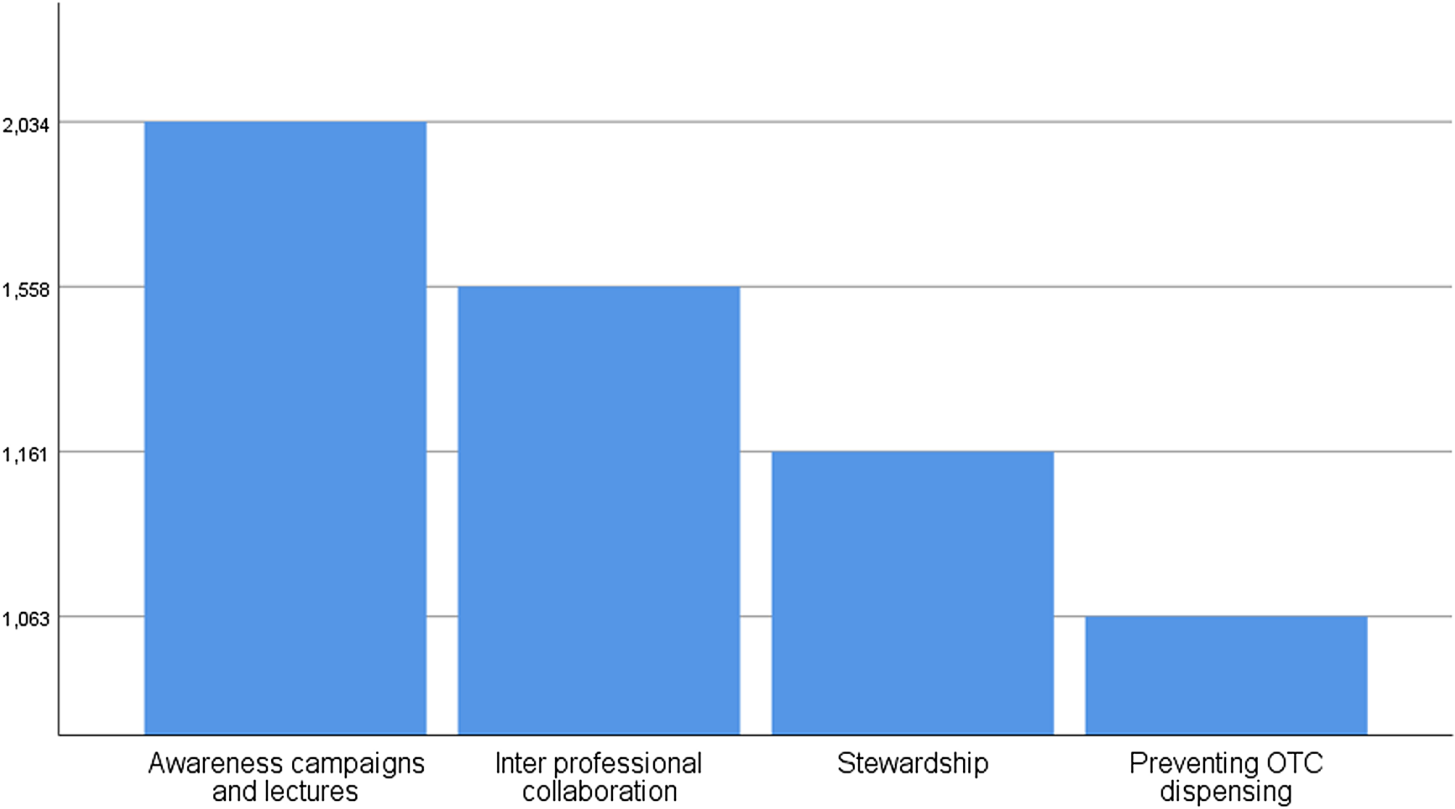
Answers to how can antimicrobial resistance be reduced

Table 4 shows the attitude of participating pharmacists. More than a third (36.8%) did not agree with the policy of not dispensing antibiotics without prescription. A percentage of 75% agree that community pharmacists are qualified to prescribe antibiotics to patients with symptoms of bacterial infections. *Also*, *more than half of pharmacists (53.9%) think that new antimicrobials cannot solve the antimicrobial resistance problem*. Nearly half the participants (45.7%) noticed an association between patients demanding antimicrobials without medical prescription and difficulty in obtaining a medical consultation. When asked, 44.8% of the respondents agreed that patients should be advised to save a portion of their antibiotic course for a different occasion, which will help them save money on their medical costs. We finally asked the participating pharmacists about how the antimicrobial resistance can be reduced; their answers are demonstrated in Fig. 4.

## Discussion

AMR poses a health crisis that affects various regions worldwide including the Middle East. To our best knowledge, this is the first study of its kind to be conducted across nineteen Arabian country. Through our study, we intended to gauge precisely how knowledgeable, responsible, and mindful pharmacists are regarding antimicrobial resistance as well as dispensing antibiotics without prescriptions. This research is groundbreaking for this region as it represents a step, towards comprehending and addressing this issue. As per our analysis, most respondents belonged to diverse community pharmacies; backed up closely behind were hospital stores along with chain ones. The level of participation exceeded expectations with an outstanding response rate standing at 91.3%, affirming their inclination towards such important issues relating to medication management systems.

Based on findings from this research indicate notable gaps exist among pharmacists’ comprehension regarding both antibiotic prescribing principles as well as associated bacterial resistance models. Our findings endorse a trend consistently observed in other independent studies conducted within the Middle Eastern areas. Similarly, and According to a survey carried out in Saudi Arabia, the majority of participants gave the incorrect response. A total of 40 community pharmacists were surveyed, and of those, about 31 (78%) had never heard of the term “antimicrobial resistance” and 32 (80%) did not understand what antibacterial resistance actually meant [21]. Another study conducted in China states that Only 9.7% of the participants had high knowledge regarding antibiotics, the majority of whom had low understanding (22.3%) or average knowledge (69.4%) [26].

An alarming discovery was made during this study as almost half of the participants (41.2%) lacked knowledge regarding the ineffectiveness of antibiotics in treating common illnesses such as colds, coughs, the flu, and fevers with temperatures lower than 38.5 °C. Similar results have been found in other recent studies. For instance, a cross- sectional study held in low and middle-income countries, including Sri Lanka revealed that 44% of pharmacist answered that “viral diseases can be treated with antibiotics” [27]. Also, a pseudo-patient study found that 66% of antibiotics is being inappropriately given to treat viral infections [28].

According to the findings of a recent investigation, it appears that community pharmacists in the countries under study commonly engage in dispensing antibiotics without requiring prescriptions (DAwP) by percentage of 75%. This finding is consistent with similar around the globe. Almaaytah et al. study demonstrated that estimated that 97.6% of Jordanian chemists have provided antibiotics without a prescription for symptoms of sore throats [10]. A study in Pakistan, where antibiotics may be purchased without a prescription from the chemist, underlined the significance of enacting rigorous rules and policies to maintain better control over the use of antibiotics [29].

Looking at the statistics, one can observe that many pharmacists do not follow proper protocols regarding antibiotic dispensation as per local guidelines. A staggering number exceeding half showcases sub-optimal practices on this front, which needs quick attention and remedial action due to its contribution towards the surge in antibiotic resistance instances across the region today.

Increased sales targets may be driving some pharmacists toward unwise dispensing practices contributing further still to antibiotic resistance. As in our study About 67.5% of the respondents agreed that refusing (DAwP) will negatively affect sales and profits. Numerous studies have shown that a significant proportion of pharmacy workers view withholding antibiotics without a prescription as problematic for their business success. A study conducted in Northern Nigeria showed that respondents’ reasons for dispensing antibiotics without prescription were “Patient will try to obtain antibiotic from another pharmacy” (32.7%) and “Fear of losing client/patient” (17.3%) [30]. The solution lies in enhanced awareness raising around responsible use of these essential medications starting with better education across all audiences involved in healthcare delivery.

On the side, Pharmacists provided some noteworthy advice in response to adult patients suffering from coughs, sore throats and runny noses according to this perceptive research. Most frequently, they recommended symptom-based OTC medication with referral to a doctor, while suggesting taking antibiotics at a much lower rate. This positive result was also found by Darwish et al., 2021. in their study they revealed that More over half (51.8%) of pharmacists stated that “patients who had a sore throat, a runny nose, or a cough would receive symptomatic medication” [10]. This suggests that pharmacists have developed an appropriate awareness around antibiotic usage which portrays promising advancement on this front.

Despite some strengths in inter-professional collaboration, data indicates ample room for enhancing communicative practices among clinicians and pharmacists alike. While just under half of all surveyed pharmacists attest to regularly communicating with prescribing physicians when confronted with uncertainty regarding antibiotic treatments, nearly 13% admit avoiding contact entirely because of negative experiences associated with physician responsiveness. This issue underscores an urgent need to foster more robust communication networks as well as increased accountability on the part of medical practitioners.

### Limitation

Although every effort was made to reassure participating pharmacists that the study was confidential, anonymous, and conducted by an academic team solely for research purposes, the Hawthorne effect could not be completely negated because some pharmacists may have behaved differently in the presence of the researchers, which could have had an impact on the outcomes. Also, one limitation of our study is the potential for convenience sample bias, as participants were recruited through a non-randomized approach, which may limit the generalizability of our findings to broader populations. In addition to that, even though a sufficient sample size was obtained for the Middle East, representation from several nations was unsatisfactory when compared to the number of pharmacists who are now employed in those nations. Another One limitation arises from potential issues inherent to Google Forms, including response bias, technical glitches, limited accessibility, data security concerns, and sampling bias, which collectively may impact the reliability and generalizability of the collected data.

## Conclusions

Our recent survey presents pertinent insights into how practicing pharmacists dispense antibiotics without prescriptions alongside their knowledge on antimicrobial resistance. Our research discovers substantive unevenness across the board relating to antibiotic prescribing principles and bacterial resistance; similarly identified trends from other relevant cross-cultural studies conducted within China/the Middle East still confirms our discoveries’ overall significance for increasing public education & awareness regarding these issues.

Generally alarming results were attained indicating a lack of understanding amongst some participating pharmacists about antibiotics’ ineffectiveness at treating viral illnesses; this exacerbated by further findings that some respondents continue to dispense them without proper prescriptions. Consequently, revealing why stricter regulations governing access controls on antibiotics dispensing are vital toward reducing excessive consumption practices.

Finally, we recommend increased collaborations between health care providers within hospitals so we can improve communication among all parties involved as well as ensuring accountability from both pharmacy staff members/medical practitioners responsible for managing patient’s treatment processes positively impacted.

Ultimately our discoveries underscore why enhancing education about responsible antibiotic use via awareness campaigns is vital to battling bacterial resistance levels that continue to rise within our communities.

### Data collection group

Abdallah ahmad Khatatbeh, Aesha L. Enairat, Ahmad Al-Farhan, Ahmed Al-Qaisi, Ahmed khairy Soliman azmy, Ahmed Naeem, Aiman Al-Touny, Bushra Muneer Rageh, Dana Alselaibi, Dina yameen Elraggal, Elnazeir Mohamed Ibrahim Mohamed Zain, Eman Alrefai, Esraa Saber Saad, Fatima A. Alkubaisi, Fatima Abd Elmonim Mohammed Abd Elkareem, Gehad Fouad, Haneen A. Mousa, Hanooafa Nasser Qaid, Heba A. Azmy, Heba Khaled Alqadasi, Hiam Al-Atnah, Hussein Kamal Seif, Kareman Ahmed Mohamed, Maha Abdellatif, Majd H. Alkhawaldeh, Manar Abdelnaser Ezzat, Marwan Badran, Milda Al Khatib, Mohamed Ahmed Ali, Mohamed Alsori, Mohamed Ashraf Rashad, Mohamed A. Elsayed, Mohamed Khaled AboSalama, Mohammad S. Abdalhadialmqadma, Mohammed Amir Rais, Mohammed T. Aliwaiti, Mohamed Mounir Gharib, Mostafa Elfrly, Mustafa Hilo Radhy, Omar farouk ismail, Raga A Elzahaf, Ragaa magdy, Raghda Rabe Hamed, Rahaf Makhlouf, Ritta Shahada, Samar Basuny shalash, Shaima’a Qaid Al-Sharaby, Shimaa A. Al-Touny, Tawfiq Abdu Ali Ali, Waheeb Ali Al-Garadi, Wessal Hassan Ali, Wisam A Hasan, Yasmine Mousa.

## Data Availability

Data is accessed only by the research management team to ensure data confidentiality.
